# HOME HEALTH AGENCY STAFF SAFETY: EXPERIENCES FROM THE FRONTLINES

**DOI:** 10.1093/geroni/igae098.1816

**Published:** 2024-12-31

**Authors:** Natalie Leland, Amanda Shore, Julia Lam, Rachel Prusynski, Debra Saliba, Tracy Mroz

**Affiliations:** University of Pittsburgh, Pittsburgh, Pennsylvania, United States; University of Pittsburgh, Pittsburgh, Pennsylvania, United States; University of Pittsburgh, Pittsburgh, Pennsylvania, United States; University of Washington, Seattle, Washington, United States; UCLA, Los Angeles, California, United States; University of Washington, Seattle, Washington, United States

## Abstract

Prior evidence has documented workplace violence among home healthcare agency (HHA) staff. Building on prior evidence, we examined organizational strategies for fostering HHA staff safety and frontline staff experiences before and after the emergence of the COVID-19 pandemic. We engaged a purposive national sample of 102 HHA leaders in virtual one-on-one semi structured interviews completed between November 2020 and December 2022. We then interviewed a subsample of HHA frontline staff (n=12) between April 2023-March 2024. Using a multiple case study design, we conducted a thematic analysis. Five emergent themes captured organizational efforts as well as staff experiences navigating workplace violence. Leadership (a) contrasted HHA safety environment with institutional healthcare, which impacts organizational policies (e.g., gun safety, compliance with COVID-19 infection control policies) and (b) described financial investment in staff safety (e.g., employ escorts to travel with HHA staff). Frontline staff themes included navigating (c) physical and verbally volatile situations during the HHA care encounter (e.g., requesting compliance with HHA’s PPE policy when HHA staff were in the home), (d) community safety, and (e) HHA safety policies and processes. Results highlight the complexity of HHA service delivery, which existed before COVID-19 and was exacerbated as the pandemic evolved. These experiences emphasize the importance of a multi-modal approach to a HHA culture of staff safety (e.g., policies, financial resources). Further, these findings suggest a need for policies that address the operational investments needed to foster an environment of safety.

